# Histological prediction and choice of the best resection strategy in front of a colorectal lesion > 2 cm: prospective comparison of endoscopic characterization, non-targeted and targeted biopsies

**DOI:** 10.1007/s00464-024-11501-7

**Published:** 2025-01-08

**Authors:** Pierre Lafeuille, Emilien Daire, Jérôme Rivory, Florian Rostain, Jean-Christophe Saurin, Thomas Lambin, Frédéric Moll, Fabien Subtil, Tanguy Fenouil, Jérémie Jacques, Mathieu Pioche

**Affiliations:** 1 Department of Gastroenterology and Endoscopy, Edouard Herriot Hospital, 69437 Lyon, France; 2 Service de Biostatistique, Hospices Civils de Lyon, Lyon, France; 3 Laboratoire de Biométrie Et Biologie Evolutive UMR 5558, Université de Lyon, Université Lyon 1, CNRS, Villeurbanne, France; 4 Institute of Pathology Est, Hospices Civils de Lyon, Lyon, France; 5 Department of Gastroenterology and Endoscopy, Dupuytren University Hospital, Limoges, France

**Keywords:** Colorectal lesion, Endoscopic characterization, Histological prediction, Biopsies, Endoscopic mucosal resection, Endoscopic submucosal dissection

## Abstract

**Background:**

Accurate endoscopic characterization of colorectal lesions is essential to predict histology and select the best treatment strategy but remains very difficult. Instead of the recommended endoscopic characterization, many gastroenterologists routinely perform biopsies of the lesion to propose endoscopic resection with or without R0 intent. The aim of this study was to determine which of endoscopic characterization or biopsies, either targeted (TB) or non-targeted (NTB), is the most effective to determine the best treatment strategy for colorectal neoplasia > 2 cm.

**Methods:**

We prospectively assessed the best strategy between endoscopic characterization and targeted or non-targeted biopsies, so that the proposed resection technique offered a level of quality of tumor resection adapted to the definitive histology of the lesion on R0-resected specimen.

**Results:**

84 patients with 88 lesions were included. “Adequate treatment” was proposed by endoscopic characterization in 52.3 to 70.5% of cases, “under treatment” in 2.3 to 9.1% and “over treatment” in 20.5 to 45.5%. Two severe events were recorded. “Adequate treatment” was proposed by TB and NTB in respectively 72.7 and 69.3% of cases, “under treatment” in respectively 27.3 and 30.7% and no case of “over treatment” was reported. TB and NTB were ineffective to evaluate the depth of invasion in the submucosa and to differentiate superficial invasive from deep invasive adenocarcinomas.

**Conclusions:**

Biopsies-based strategies are unable to predict depth of cancer invasion and could be associated with a risk of under treatment of large colorectal lesions in near a third of the cases compared to only around 5% with endoscopic characterization. Endoscopic characterization could lead to over treatment, but mainly by endoscopic submucosal dissection with low morbidity. Characterization with the CONECCT classification could decrease the risk of under treatment and avoid surgeries for non-malignant colorectal lesions. Other endoscopic criteria should be determined to better characterize colorectal lesions and to improve the best adapted treatment for each lesion.

**Graphical abstract:**

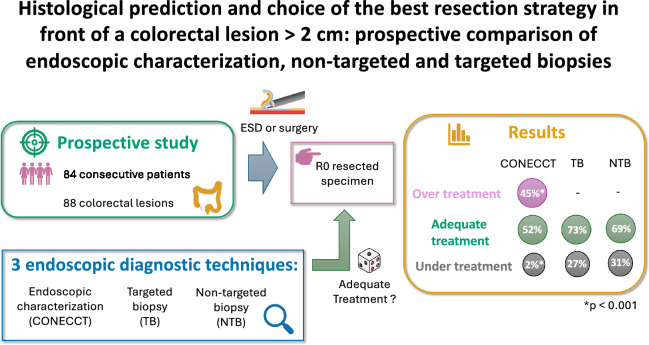

Colorectal cancer has become a public health priority considering its increased prevalence and high mortality rate when diagnosed late [1]. Therefore, many countries have promoted colorectal cancer screening programs leading to an increasing detection of advanced but also superficial lesions [1]. ESGE (European Society of Gastrointestinal Endoscopy) guidelines states that most of those colorectal superficial lesions can be removed in a curative way by standard polypectomy and/or by Endoscopic Mucosal Resection (EMR). Endoscopic Submucosal Dissection (ESD) can be considered for removal of lesions with high suspicion of superficial submucosal invasion especially for lesions larger than 20 mm. Curative resection is determined by the association of several pathological criteria after microscopic assessment: low-grade adenocarcinoma (G1/G2), depth of invasion ≤ 1000 μm in the submucosa or sm1 adenocarcinoma, no lymphovascular invasion (LVI) nor significant budding (no Bd2/Bd3), and free of cancer horizontal and vertical margins also stated as R0 resection [2]. Unfortunately, those criteria can only be analyzed on the endoscopic resection specimen. The challenge is then to indirectly predict these pathological criteria before endoscopic treatment using endoscopic diagnostic tools to propose the most adequate treatment for each lesion type: standard polypectomy and EMR for benign or intra-epithelial lesions, ESD or surgery for invasive adenocarcinoma. Many characterization criteria have been described in several classifications, such as Paris [3], Kudo [4] and Sano [5] classifications. More recently, the CONECCT classification (Fig. 1) was created as a combination of the different validated criteria commonly used in a single table to simplify the prediction process [6–8].

**Fig. 1:**
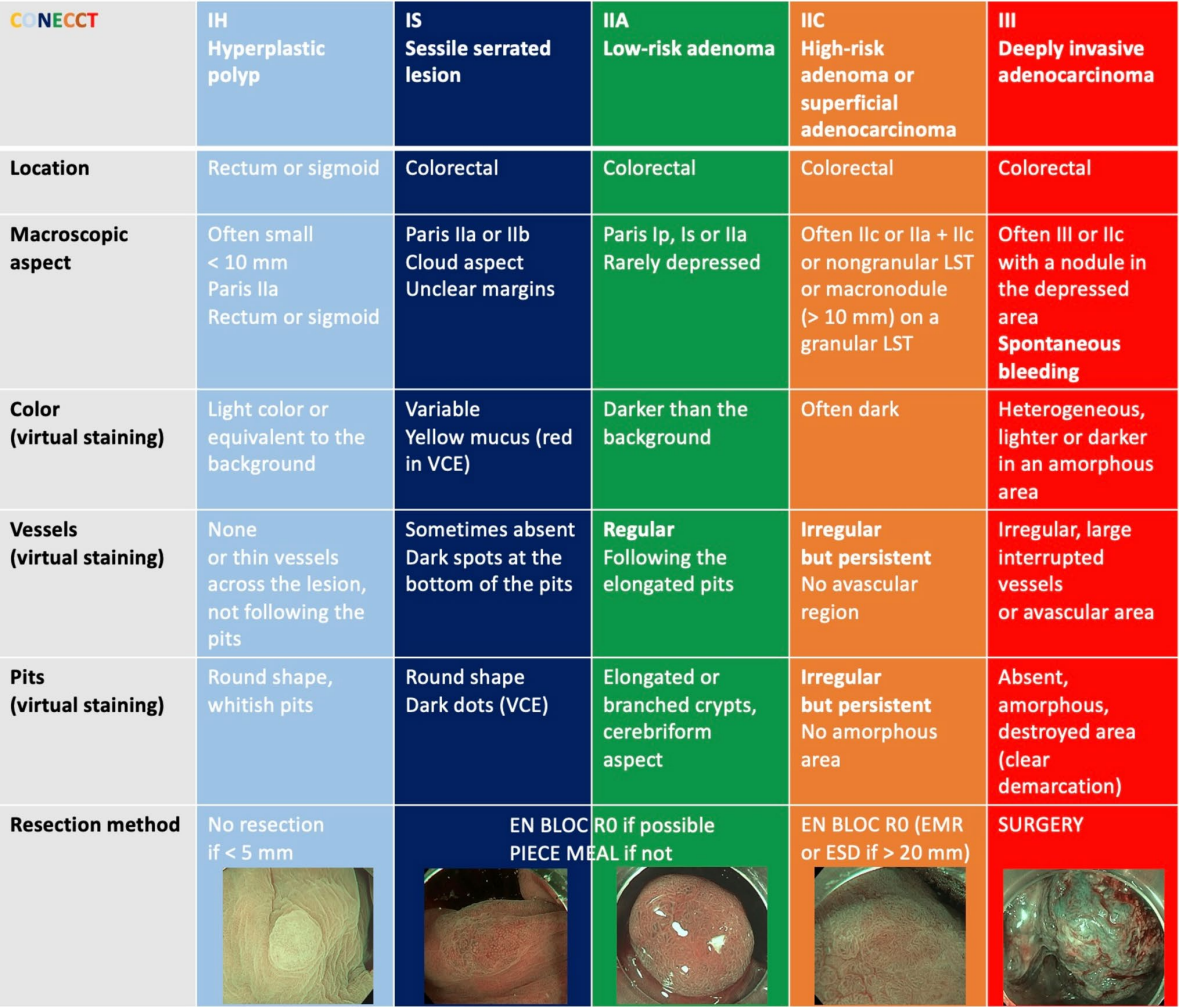
The CONECCT Classification. EMR, Endoscopic Mucosal Resection; ESD, Endoscopic Submucosal Dissection; LST, Laterally Spreading Tumor; VCE, Virtual Chromoendoscopy

In our experience, although endoscopic characterization is effective for predicting depth of invasion of colorectal neoplasia to choose the most appropriate resection technique, few gastroenterologists are trained and many of them routinely perform biopsies of the lesion to guide the resection strategy. Depending on the presence or absence of cancer on biopsies, endoscopic or surgical resection is proposed leading to frequent surgeries for non-malignant colorectal lesions [9, 10] with avoidable morbidity [11]. Furthermore, such a strategy could misclassify patient’s colorectal lesions considering that the topographical distribution of different histological features is not uniform throughout a colorectal neoplasia with both malignant and premalignant zones coexisting, especially for large lesions [11]. Moreover, biopsies appear to be too much superficial to be effective for predicting depth of invasion.

We conducted a prospective observational cohort study to determine which of endoscopic characterization or biopsies, either targeted (TB) or non-targeted (NTB), is the most effective technique to determine the best treatment strategy for colorectal neoplasia > 2 cm.

## Patients and methods

### Study design

We conducted a prospective observational cohort study at our tertiary referral center in France, including all consecutive patients referred to our center for the endoscopic resection of a colorectal neoplasia > 2 cm, between January 2019 and January 2020.

Patients aged of ≥ 18 years old without previous history of endoscopic resection nor resection attempt were included. Information on lesion characteristics, such as biopsies or images from previous endoscopy reports, was not provided to the physician in charge of endoscopic characterization and resection.

Patients were excluded in case of underlying inflammatory bowel disease, or when the specimen was not resected en bloc R0 in case of adenocarcinoma. When ESD was not curative and the histological analysis reported deep invasive adenocarcinoma, the patient was included considering that the histological analysis could not be underestimated. In case of focal positive horizontal margin with only SSL (Sessile Serrated Lesion) or low-grade dysplasia, the lesion was not excluded considering that the histological analysis was of good quality without loss of information.

The Ethics Committee of Hospices Civils de Lyon approved the study, and all patients gave informed consent before their procedures. The study was declared on the national clinical trial register with the number NCT04482491.

### Colonoscopy procedures

All colonoscopies were performed by three senior endoscopists (M.P., J.R., F.R.), experienced in advanced diagnosis and ESD (over 300 procedures each) with the patient under general anesthesia and using CO2 insufflation. Optical characterization of lesions was performed using high-definition white light endoscopy followed by close-up examination assisted by virtual chromoendoscopy, with or without magnification, using Olympus CF-HQ190 L/I colonoscopes (Olympus, Tokyo, Japan).

### Resection techniques used to obtain high-quality histological assessment

In this study, to avoid missing information, we aimed to obtain for all colorectal neoplasia > 2 cm included a high-quality histological assessment [7]:

- For lesions without any suspicion of deep invasive component (lesions > 2 cm without any suspicion of deep invasive component and lesions with a tiny area < 5 mm of amorphous pit or vascular patterns as proposed by Japanese guidelines [12]), an ESD was performed with an en bloc R0 intent.
- For lesions with high suspicion of deep invasive component (lesions with a large amorphous area > 5 mm), a surgery was performed after the following endoscopic biopsy protocol to achieve a surgical R0 resection with lymphadenectomy.

### Biopsy procedures

When ESD was feasible, biopsies were performed after the end of the procedure, on the specimen itself after stretching it on cork, to avoid any damage or bleeding that could have interfered with the resection leading to an over risk for the patient. If an area appeared suspicious of focal invasion during endoscopic characterization, a targeted biopsy (TB) was done on this area, after recognition of the area on the specimen itself, guided with the endoscope using white light imaging (WLI) and/or virtual chromoendoscopy. A non-targeted biopsy (NTB) was then randomly performed anywhere in the lesion. In the case when no area was suspicious of invasion, only a single NTB was performed.

When a surgical resection was proposed, TB and NTB were performed in the same way, directly after endoscopic characterization in the colon or rectum.

### Histological examination

Histopathological examination was carried out by two different expert digestive pathologists, double blinded from the result of the other analysis and the endoscopic prediction, according to the Vienna [13] and TNM [14] classifications. For biopsies specimen, as depth of invasion could not be estimated, an “infiltrating aspect” was considered as deep invasive adenocarcinoma.

### Study primary objective

The primary objective was the evaluation of the best strategy between endoscopic characterization and targeted or non-targeted biopsies, so that the proposed resection technique offered a level of quality of tumor resection adapted to the definitive histology of the lesion on resected specimen, defined by the following:

- Resection without R0 intent (piece meal) for sessile serrated lesion (SSL), low-grade (LGD) and high-grade (HGD) dysplastic adenoma (CONECCT IS or IIA, Kudo II, III or IV, Sano I or II),
- En Bloc R0 resection (ESD) for intramucosal adenocarcinoma, superficial submucosal adenocarcinoma with < 1000 μm submucosal invasion (CONECCT IIC, Kudo Vi, Sano IIIA),
- Surgical resection with R0 intent for deep invasive lesions: deep submucosal adenocarcinoma with > 1000 μm submucosal invasion, intramuscular or deeper T2-T3 cancer (CONECCT III, Kudo Vn, Sano IIIB, infiltrating aspect without any precision at the histology report of biopsies).

“Adequate treatment” was defined when the proposed resection technique offered a level of quality of tumor resection adapted to the definitive histology of the lesion, “under treatment” when it led to an insufficient level of quality of tumor resection and “over treatment” when an overly invasive resection technique would have been proposed.

### Study secondary objectives

Secondary objectives were the diagnostic accuracy to predict histology (sensitivity Se and specificity Sp) of endoscopic characterization, TB and NTB, the evaluation of adequacy of proposed treatment by biopsies with final histology if we consider that the presence of cancer whatever its depth would lead to surgical treatment, and adverse events occurring during or after endoscopic and surgical resections.

### Data collection

The data collected were patient demographics including sex and age at the time of colonoscopy; lesion characteristics: location, size, morphology, classification according to Paris, Kudo, Sano, and CONECCT classifications, histology of TB, NTB and final histology after ESD or surgical resection.

### Statistical analysis

Continuous variables were presented as mean ± standard deviation or median with the first and the third quartile. Categorical variables were presented as numbers and percentages. A chi-square test was used to compare adequacy of proposed treatment with final histology. Diagnostic accuracy was assessed by sensitivity and specificity. A Mc Nemar test was used to compare sensitivity and specificity between paired groups of techniques. A p-value of less than 0.05 was considered significant.

## Results

### Study population

**Fig. 2:**
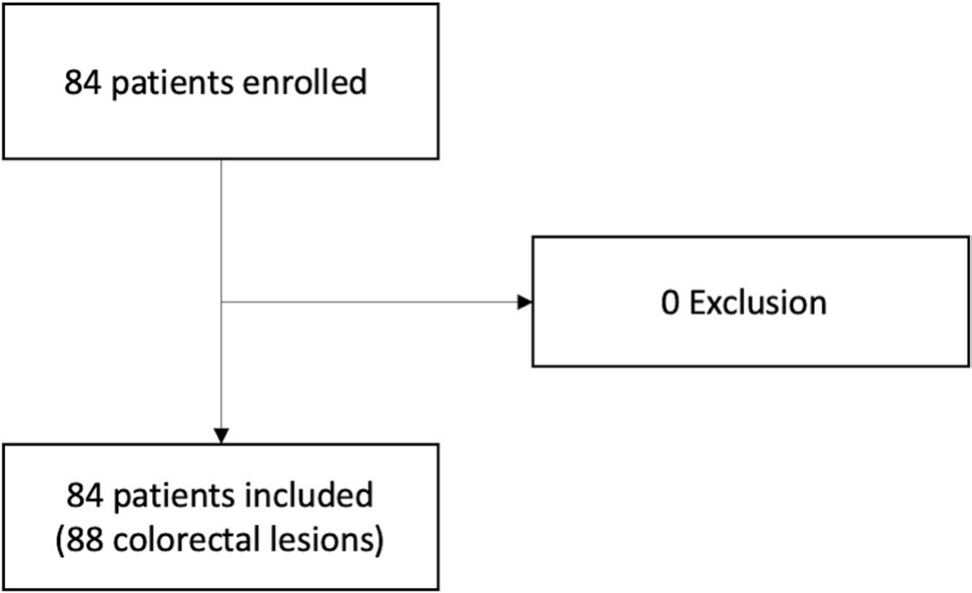
Flow-chart of the study

We prospectively included 84 patients with 88 colorectal lesions, median age 69 years (range, 60–74); 51 men and 33 women (Fig. 2). Patients and colorectal lesions characteristics are presented in Tables 1, 2, respectively.

**Table 1:** Characteristics of patients

Characteristic	
Patients, *n*	84
Gender, *n* (%)	
Male	51 (60.7)
Female	33 (39.3)
Age at diagnosis, y	
Median (range)	69 (60–74)

**Table 2:** Characteristics of colorectal lesions

Characteristic	
Lesions, *n*	88
Location, *n* (%)	
Caecum	22 (25)
Right colon	24 (27.3)
Transverse colon	7 (8.0)
Left colon	6 (6.8)
Sigmoid	15 (17.0)
Rectum	12 (13.6)
na	2 (2.3)
Macroscopic type, n (%)	
Polypoid	3 (3.4)
Granular homogeneous LST	18 (20.4)
Granular mixt LST	33 (37.5)
Granular LST with depressed area	8 (9.1)
Nodular LST	1 (1.1)
Flat non granular LST	9 (10.2)
Pseudodepressed non granular LST	6 (6.8)
Pseudodepressed non granular LST with macronodule	1 (1.1)
na	9 (10.2)
Macronodule > 1 cm, *n* (%)	
Yes	35 (39.8)
No	53 (60.2)
Paris classification, *n* (%)	
Ip	2 (2.3)
Is	10 (11.4)
Is-IIa	13 (14.8)
Is-IIa-IIc	1 (1.1)
Is-IIc	13 (14.8)
Is-III	1 (1.1)
IIa	21 (23.9)
IIa-Is	8 (9.1)
IIa-IIc	11 (12.5)
IIc	6 (6.8)
III	2 (2.3)
Kudo classification, *n* (%)	
I	1 (1.1)
IIO	5 (5.7)
IV	39 (44.3)
Vi	21 (23.9)
Vn	22 (25.0)
Sano classification, *n* (%)	
I	6 (6.8)
II	35 (39.8)
IIIA	25 (28.4)
IIIB	22 (25.0)
CONECCT classification, *n *(%)	
IS	6 (6.8)
IIA	11 (12.5)
IIC	47 (53.4)
IIC + IS	2 (2.3)
III	22 (25.0)
Histology, *n* (%)	
Sessile serrated lesion	6 (6.8)
Low-grade dysplastic adenoma (Vienna 4.1)	8 (9.1)
High-grade dysplastic adenoma (Vienna 4.1)	38 (43.2)
Intramucosal adenocarcinoma (Vienna 4.4)	14 (15.9)
Superficial submucosal adenocarcinoma (< 1000 µm)	3 (3.4)
Deep submucosal adenocarcinoma (> 1000 µm), intramuscular or deeper cancer	19 (21.6)

*LST* Laterally Spreading Tumor *na* not attributed

### Histology of resected lesions

Histology revealed 6/88 (6.8%) sessile serrated lesions (SSL) without any dysplasia, 8/88 (9.1%) LGD adenomas, 38/88 (43.2%) HGD adenomas, 14/88 (15.9%) intramucosal adenocarcinomas, 3/88 (3.4%) superficial submucosal adenocarcinomas with < 1000 μm submucosal invasion and 19/88 (21.6%) deep submucosal adenocarcinomas with > 1000 μm submucosal invasion, intramuscular or deeper T2-T3 cancers (Table 2).

## Endoscopic characterization strategy with CONNECT classification

### Diagnostic accuracy

The characterization with the CONECCT classification strategy classified SSL as CONECCT IS in 6/6 (100%) cases (Se = 100.0% and Sp = 100.0%). Adenomas were classified as CONECCT IIA in 10/46 (21.7%) cases and CONECCT IIC in 36/46 (78.3%) cases. LGD adenomas were classified as CONECCT IIA in 3/8 (37.5%) cases and CONECCT IIC in 5/8 (62.5%) cases. HGD adenomas were classified as CONECCT IIA in 7/38 (18.4%) cases and CONECCT IIC in 31/38 (81.6%) cases. Diagnostic accuracy for adenomas was as follow: Se = 21.7%, Sp = 97.6%. Adenocarcinomas were classified as CONECCT IIC or CONECCT III in 35/36 (97.2%) cases. 12/17 (70.6%) intramucosal or superficial invasive adenocarcinomas were classified as CONECCT IIC (1/17 (5.9%) as CONECCT IIA and 4/17 (23.5%) as CONECCT III). 18/19 (94.7%) deep invasive adenocarcinomas were classified as CONECCT III (1/19 (5.3%) as CONECCT IIC). Diagnostic accuracy to distinguish benign from malignant lesions was as follow: Se = 30.8%, Sp = 97.2%. Diagnostic accuracy to distinguish deep invasive adenocarcinomas from other lesions was as follow: Se = 94.7%, Sp = 94.2%. (Tables 3, 9 and 10).

**Table 3:** Evaluation of the endoscopic characterization strategy to predict final histology (CONECCT)

	Histology of resected lesions						
CONECCT	SSL	LGD adenoma	HGD adenoma	Intramucosal adenocarcinoma	Superficial invasive adenocarcinoma	Deep invasive adenocarcinoma	Total
IS	6 (100.0%)	0	0	0	0	0	6 (6.8%)
IIA	0	3 (37.5%)	7 (18.4%)	1 (7.1%)	0	0	11 (12.5%)
IIC	0	5 (62.5%)	31 (81.6%)	12 (85.8%)	0 (0.0%)	1 (5.2%)	49 (55.7%)
III	0	0	0	1 (7.1%)	3 (100.0%)	18 (94.7%)	22 (25.0%)
Total	6 (6.8%)	8 (9.1%)	38 (43.2%)	14 (15.9%)	3 (3.4%)	19 (21.6%)	88 (100.0%)

*SSL* Sessile serrated lesion, *LGD* Low-grade dysplasia, *HGD* High-grade dysplasia

### Adequacy of proposed treatment with final histology

“Adequate treatment” was obtained in 46/88 (52.3%; *p* = 0.042) cases, “under treatment” in 2/88 (2.3%; *p* < 0.001) cases with one case of piece meal EMR (instead of ESD) for intramucosal adenocarcinoma and one case of ESD (instead of surgery) for deep invasive adenocarcinoma, and “over treatment” in 40/88 (45.4%; *p* < 0.001) cases with 36 cases of ESD (instead of piece meal) for adenomatous lesions classified CONECCT IIC instead of IIA, and 4 cases of surgery (instead of ESD) for one case of intramucosal adenocarcinoma and three superficial invasive adenocarcinomas classified CONECCT III instead of IIC. The number of avoidable surgeries using the CONECCT classification was 4/88 (4.5%) (Table 4).

**Table 4:** Adequacy of proposed treatment with final histology

	Endoscopic characterization			TB	NTB	*p*-value
	CONECCT	Kudo	Sano			
Over treatment, *n* (%)	40/88 (45.5)	18/88 (20.5)	21/88 (23.9)	0	0	< 0.001
Adequate treatment, *n* (%)	46/88 (52.3)	62/88 (70.5)	60/88 (68.2)	64/88 (72.7)	61/88 (69.3)	0.042
Under treatment, *n* (%)	2/88 (2.3)	8/88 (9.1)	7/88 (8.0)	24/88 (27.3)	27/88 (30.7)	< 0.001

*TB* Targeted biopsy, *NTB* Non-targeted biopsy

## Endoscopic characterization strategy with Kudo classification

### Diagnostic accuracy

The characterization with the Kudo classification strategy classified SSL as Kudo I and IIO in 1/6 (16.7%) and 5/6 (83.3%) cases, respectively (Se = 100.0% and Sp = 100.0%). Adenomas were classified as Kudo IV in 32/46 (69.6%) cases and Kudo Vi in 14/46 (30.4%) cases. LGD adenomas were classified as Kudo IV in 7/8 (87.5%) cases and Kudo Vi in 1/8 (12.5%) cases. HGD adenomas were classified as Kudo IV in 25/38 (65.8%) cases and Kudo Vi in 13/38 (34.2%) cases. Diagnostic accuracy for adenomas was as follow: Se = 69.6%, Sp = 83.3%. Adenocarcinomas were classified as Kudo Vi or Kudo Vn in 29/36 (80.6%) cases. 6/17 (35.3%) intramucosal or superficial invasive adenocarcinomas were classified as Kudo Vi (7/17 (41.2%) as Kudo IV and 4/17 (23.5%) as Kudo Vn). 18/19 (94.7%) deep invasive adenocarcinomas were classified as Kudo Vn (1/19 (5.3%) as Kudo Vi). Diagnostic accuracy to distinguish benign from malignant lesions was as follow: Se = 73,1%, Sp = 80,6%. Diagnostic accuracy to distinguish deep invasive adenocarcinomas from other lesions was as follow: Se 94.7%, Sp 94.2%. (Tables 5, 9 and 10).

**Table 5:** Evaluation of the endoscopic characterization strategy to predict final histology (Kudo)

	Histology of resected lesions						
Kudo	SSL	LGD adenoma	HGD adenoma	Intramucosal adenocarcinoma	Superficial invasive adenocarcinoma	Deep invasive adenocarcinoma	Total
I	1 (16.7%)	0	0	0	0	0	1 (1.1%)
II	0	0	0	0	0	0	0
IIO	5 (83.3%)	0	0	0	0	0	5 (5.7%)
III	0	0	0	0	0	0	0
IV	0	7 (87.5%)	25 (65.8%)	7 (50.0%)	0	0	39 (44.3%)
Vi	0	1 (12.5%)	13 (34.2%)	6 (42.9%)	0	1 (5.3%)	21 (23.9%)
Vn	0	0	0	1 (7.1%)	3 (100.0%)	18 (94.7%)	22 (25.0%)
Total	6 (6.8%)	8 (9.1%)	38 (43.2%)	14 (15.9%)	3 (3.4%)	19 (21.6%)	88 (100.0%)

*SSL* Sessile serrated lesion, *LGD* Low-grade dysplasia, *HGD* High-grade dysplasia

### Adequacy of proposed treatment with final histology

“Adequate treatment” was obtained in 62/88 (70.4%; *p* = 0.042) cases, “under treatment” in 8/88 (9.1%; *p* < 0.001) cases with 7 cases of piece meal EMR (instead of ESD) for intramucosal adenocarcinomas and one case of ESD (instead of surgery) for deep invasive adenocarcinoma, and “over treatment” in 18/88 (20.4%; *p* < 0.001) cases with 14 cases of ESD (instead of piece meal) for adenomatous lesions classified Kudo Vi instead of Kudo IV and 4 cases of surgery (instead of ESD) for one case of intramucosal adenocarcinoma and three cases of superficial invasive adenocarcinomas classified Kudo Vn instead of Kudo Vi. The number of avoidable surgeries using the Kudo classification was 4/88 (4.5%) (Table 4).

## Endoscopic characterization strategy with Sano classification

### Diagnostic accuracy

The characterization with the Sano classification strategy classified SSL as Sano I in 6/6 (100%) cases (Se = 100.0% and Sp = 100.0%). Adenomas were classified as Sano II in 29/46 (63.0%) cases and Sano IIIA in 17/46 (37.0%) cases. LGD adenomas were classified as Sano II in 6/8 (75.0%) cases and Sano IIIA in 2/8 (25.0%) cases. HGD adenomas were classified as Sano II in 23/38 (60.5%) cases and Sano IIIA in 15/38 (39.5%) cases. Diagnostic accuracy for adenomas was as follow: Se = 63.0%, Sp = 85.7%. Adenocarcinomas were classified as Sano IIIA or Sano IIIB in 30/36 (83.3%) cases. 7/17 (41.2%) intramucosal or superficial invasive adenocarcinomas were classified as Sano IIIA (6/17 (35.3%) as Sano II and 4/17 (23.5%) as Sano IIIB). 18/19 (94.7%) deep invasive adenocarcinomas were classified as Sano IIIB (1/19 (5.3%) as Sano IIIA). Diagnostic accuracy to distinguish benign from malignant lesions was as follow: Se = 67.3% Sp = 83.3%. Diagnostic accuracy to distinguish deep invasive adenocarcinomas from other lesions was as follow: Se = 94.7%, Sp = 94.2%. (Tables 6, 9 and 10).

**Table 6:** Evaluation of the endoscopic characterization strategy to predict final histology (Sano)

	Histology of resected lesions						
Sano	SSL	LGD adenoma	HGD adenoma	Intramucosal adenocarcinoma	Superficial invasive adenocarcinoma	Deep invasive adenocarcinoma	Total
I	6 (100%)	0	0	0	0	0	6 (6.8%)
II	0	6 (75.0%)	23 (60.5%)	6 (42.9%)	0	0	35 (39.8%)
IIIA	0	2 (25.0%)	15 (39.5%)	7 (50.0%)	0	1 (5.3%)	25 (28.4%)
IIIB	0	0	0	1 (7.1%)	3 (100.0%)	18 (94.7%)	22 (25.0%)
Total	6 (6.8%)	8 (9.1%)	38 (43.2%)	14 (15.9%)	3 (3.4%)	19 (21.6%)	88 (100.0%)

*SSL* Sessile serrated lesion, *LGD* Low-grade dysplasia, *HGD* High-grade dysplasia

### Adequacy of proposed treatment with final histology

“Adequate treatment” was obtained in 60/88 (68.2%; *p* = 0.042) cases, “under treatment” in 7/88 (7.9%; *p* < 0.001) cases with 6 cases of piece meal (instead of ESD) for intramucosal adenocarcinomas and one case of ESD (instead of surgery) for deep invasive adenocarcinoma, and “over treatment” in 21/88 (23.9%; *p* < 0.001) cases with 17 cases of ESD (instead of piece meal) for adenomatous lesions classified Sano IIIA instead of Sano II and 4 cases of surgery (instead of ESD) for one case of intramucosal adenocarcinoma and three cases of superficial invasive adenocarcinomas classified Sano IIIB instead of Sano IIIA. The number of avoidable surgeries using the Sano classification was 4/88 (4.5%) (Table 4).

## TB strategy

### Diagnostic accuracy

The TB strategy diagnosed SSL in 6/6 (100%) cases (Se = 100.0% and Sp = 100.0%). TB diagnosed adenomas (LGD or HGD) in 46/46 (100%) cases, but HGD adenomas were misdiagnosed in LGD adenomas in 13/38 (34.2%) cases (Se = 100.0% and Sp = 66.7%). TB diagnosed adenocarcinomas in 22/36 cases (61.1%), 10/36 (27.8%) were misdiagnosed in HDG adenomas and 4/36 (11.1%) in LGD adenomas. TB diagnosed intramucosal adenocarcinomas in 2/14 (14.3%) cases. All 8 infiltrating aspects were deep invasive adenocarcinomas. Diagnostic accuracy of TB to distinguish benign from malignant lesions was as follow: Se = 100.0%, Sp = 61.1%. Diagnostic accuracy of TB to distinguish deep invasive adenocarcinomas from other lesions was as follow: Se = 42.1%, Sp = 100%. (Tables 7, 9 and 10).

**Table 7:** Evaluation of the TB strategy to predict final histology

	Histology of resected lesions						
TB	SSL	LGD adenoma	HGD adenoma	Intramucosal adenocarcinoma	Superficial invasive adenocarcinoma	Deep invasive adenocarcinoma	Total
SSL	6 (100.0%)	0	0	0	0	0	6 (6.8%)
LGD adenoma	0	8 (100.0%)	13 (34.2%)	4 (28.6%)	0	0	25 (28.4%)
HGD adenoma	0	0	25 (65.8%)	8 (57.1%)	1 (33.3%)	1 (5.3%)	35 (39.8%)
Intramucosal adenocarcinoma	0	0	0	2 (14.3%)	2 (66.7%)	10 (52.6%)	14 (15.9%)
Inflitrating aspect	0	0	0	0	0	8 (42.1%)	8 (9.1%)
Total	6 (6.8%)	8 (9.1%)	38 (43.2%)	14 (15.9%)	3 (3.4%)	19 (21.6%)	88 (100.0%)

*TB* Targeted biopsy, *SSL* Sessile serrated lesion, *LGD* Low-grade dysplasia, *HGD* High-grade dysplasia

### Adequacy of proposed treatment with final histology

“Adequate treatment” was obtained in 64/88 (72.7%; *p* = 0.042) cases, “under treatment” in 24/88 (27.3%; *p* < 0.001) cases with 14 cases of piece meal (instead of ESD for 13 cases and surgery for 1 case) for 12 intramucosal adenocarcinomas, one superficial invasive adenocarcinoma and one deep invasive adenocarcinoma, and 10 cases of ESD (instead of surgery) for deep invasive adenocarcinomas. There was no case of “over treatment” and consequently no case of avoidable surgery (Table 4).

If we consider that the presence of cancer whatever its depth would lead to surgical treatment, “adequate treatment” would be obtained in 70/88 (79.5%) cases, “under treatment” in 14/88 (15.9%) cases with 14 cases of piece meal (instead of ESD for 13 cases and surgery for 1 case) for 12 intramucosal adenocarcinomas, one superficial invasive adenocarcinoma and one deep invasive adenocarcinoma and “over treatment” in 4/88 (4.5%) cases with 4 cases of surgery instead of ESD for 2 intramucosal adenocarcinomas and 2 superficial invasive adenocarcinomas. The number of avoidable surgeries would have been 4/88 (4.5%).

## NTB strategy

### Diagnostic accuracy

The NTB strategy diagnosed SSL in 6/6 (100%) cases (Se = 100.0% and Sp = 100.0%). NTB diagnosed adenomas (LGD or HGD) in 46/46 (100%) cases, but HGD adenomas were misdiagnosed in LGD adenomas in 17/38 (44.7%) cases (Se = 100.0% and Sp = 50.0%). NTB diagnosed adenocarcinomas in 15/36 cases (41.7%), 13/36 (36.1%) were misdiagnosed in HDG adenomas and 8/36 (22.2%) in LGD adenomas. NTB diagnosed intramucosal adenocarcinomas in 2/14 (14.3%) cases. All 6 infiltrating aspects were deep invasive adenocarcinomas. Diagnostic accuracy of NTB to distinguish benign lesions (SSL, adenomas) from malignant lesions (intramucosal, superficial invasive or deep invasive adenocarcinomas) was as follow: Se = 100.0%, Sp = 41.7%. Diagnostic accuracy of NTB to distinguish deep invasive adenocarcinomas from other lesions was as follow: Se = 31.6%, Sp = 100.0% (Tables 8, 9 and 10).

**Table 8:** Evaluation of the NTB strategy to predict final histology

	Histology of resected lesions						
NTB	SSL	LGD adenoma	HGD adenoma	Intramucosal adenocarcinoma	Superficial invasive adenocarcinoma	Deep invasive adenocarcinoma	Total
SSL	6 (100.0%)	0	0	0	0	0	6 (6.8%)
LGD adenoma	0	8 (100.0%)	17 (44.7%)	4 (28.6%)	1 (33.3%)	3 (15.7%)	33 (37.5%)
HGD adenoma	0	0	21 (55.3%)	8 (57.1%)	1 (33.3%)	4 (21.1%)	34 (38.6%)
Intramucosal adenocarcinoma	0	0	0	2 (14.3%)	1 (33.3%)	6 (31.6%)	9 (17,1%)
Infiltrating aspect	0	0	0	0	0	6 (31.6%)	6 (6.8%)
Total	6 (6,8%)	8 (9,1%)	38 (43,2%)	14 (15,9%)	3 (3,4%)	19 (21,6%)	88 (100.0%)

*NTB* Non-targeted biopsy, *SSL* Sessile serrated lesion, *LGD* Low-grade dysplasia, *HGD* High-grade dysplasia

**Table 9:** Sensibility and specificity of NTB, TB and endoscopic characterization to predict histology of the resected lesions

		Test performance	
Aim of the test		Sensibility	Specificity
**To predict SSL**	NTB	100.0%	100.0%
	TB	100.0%	100.0%
	CONECCT	100.0%	100.0%
	Kudo	100.0%	100.0%
	Sano	100.0%	100.0%
**To predict adenoma**	NTB	100.0%	50.0%
	TB	100.0%	66.7%
	CONECCT	21.7%	97.6%
	Kudo	69.6%	83.3%
	Sano	63.0%	85.7%
**To predict benign from malignant lesions**	NTB	100.0%	41.7%
	TB	100.0%	61.1%
	CONECCT	30.8%	97.2%
	Kudo	73.1%	80.6%
	Sano	67.3%	83.3%
**To predict deep invasive adenocarcinoma from other lesions**	NTB	31.6%	100.0%
	TB	42.1%	100.0%
	CONECCT	94.7%	94.2%
	Kudo	94.7%	94.2%
	Sano	94.7%	94.2%

*SSL* Sessile serrated lesion, *NTB* Non-targeted biopsy, *TB* Targeted biopsy

**Table 10:** p-value of sensibility and specificity of TB and endoscopic characterization to predict histology of the resected lesions

	*p*-value of sensibility	*p*-value of specificity
**To predict adenoma**		
CONECCT VS TB	< 0.0001	0
Kudo VS TB	0	0.02
Sano VS TB	< 0.0001	0.005
CONECCT VS Kudo	< 0.0001	0.014
CONECCT VS Sano	< 0.0001	0.025
Kudo VS Sano	0.083	0.317
**To predict benign lesion**		
CONECCT VS TB	< 0.0001	0
Kudo VS TB	0	0.02
Sano VS TB	< 0.0001	0.005
CONECCT VS Kudo	< 0.0001	0.014
CONECCT VS Sano	< 0.0001	0.025
Kudo VS Sano	0.083	0.317
**To predict deep invasive adenocarcinoma**		
CONECCT VS TB	0.002	0.046
Kudo VS TB	0.002	0.046
Sano VS TB	0.002	0.046
CONECCT VS Kudo	1	1
CONECCT VS Sano	1	1
Kudo VS Sano	1	1

*TB* Targeted biopsy

### Adequacy of proposed treatment with final histology

“Adequate treatment” was obtained in 61/88 (69.3%; *p* = 0.042) cases, “under treatment” in 27/88 (30.7%; *p* < 0.001) cases with 21 cases of piece meal (instead of ESD for 14 cases and surgery for 7 cases) for 12 intramucosal adenocarcinomas, 2 superficial invasive adenocarcinomas and 7 deep invasive adenocarcinomas, and 6 cases of ESD (instead of surgery) for deep invasive adenocarcinomas. There was no case of “over treatment” and consequently no case of avoidable surgery (Table 4).

If we consider that the presence of cancer whatever its depth would lead to surgical treatment, “adequate treatment” would have been obtained in 64/88 (72.7%) cases, “under treatment” in 21/88 (23.9%) cases with 21 cases of piece meal (instead of ESD for 14 cases and surgery for 7 cases) for 12 intramucosal adenocarcinomas, 2 superficial invasive adenocarcinomas and 7 deep invasive adenocarcinomas and “over treatment” in 3/88 (3.4%) cases with 3 cases of surgery instead of ESD for 2 intramucosal adenocarcinomas and one superficial invasive adenocarcinoma. The number of avoidable surgeries would have been 3/88 (3.4%).

## Adverse events

ESD was technically successful for 73/88 lesions (83.0%). Adverse events occurred in 14/73 (19.2%) cases, 2/73 (2.8%) of which were severe and no death was recorded. 7/73 (9.6%) lower intestinal bleeding were recorded including 1/73 (1.4%) severe hematochezia responsible for anemia and requiring transfusion, 1/73 (1.4%) case of fever with a favorable outcome after a short antibiotic management, and 6/73 (8.2%) perforations with 3/73 (4.1%) small defects recognized during ESD procedure and successfully managed by immediate endoscopic closure, 2/73 (2.7%) managed conservatively with intravenous antibiotics and 1/73 (1.4%) leading to surgical management.

Among the 19 deep invasive adenocarcinomas, 16/19 (84.2%) were treated by surgery, 2/19 (10.5%) were treated by chemotherapy and/or radiotherapy given the comorbidities and 1/19 (5.3%) could not be treated and the patient died of other comorbidities. Among the deep invasive adenocarcinomas treated by surgery, adverse events occurred in 3/16 (18.8%) cases with 1 case of localized abscess, 1 case of anastomotic fistula and 1 death from mesenteric ischemia.

## Discussion

To the best of our knowledge, this is the first prospective study to determine which of endoscopic characterization or biopsies, either targeted or non-targeted, is the most effective technique to determine the best treatment strategy for colorectal neoplasia > 2 cm.

Biopsies (NTB and TB) allowed to determine the adequate treatment in respectively 69.3% and 72.7% which is equivalent when compared to Sano and Kudo endoscopic characterization with a rate of adequate treatment of 68.2 to 70.5%, respectively. No case of over treatment was reported in the biopsies group, which contrasts with a high risk of under treatment with respectively 30.7 and 27.3% which is much higher than with endoscopic characterization and especially the CONECCT classification with only 2.3% of under treatment. More strikingly, TB misdiagnosed 12 intramucosal adenocarcinomas, one superficial invasive adenocarcinoma and one deep invasive adenocarcinoma and NTB misdiagnosed 12 intramucosal adenocarcinomas, two superficial invasive adenocarcinomas and 7 deep invasive adenocarcinomas as adenomas. These discrepancies explain the high rate of under treatment in the biopsies group since 2/14 lesions would have been resected without R0 intent while it was needed. A study evaluated that the discrepancy rate between endoscopic biopsies and final histology of resected colorectal lesions was 10% with 7% under treatment, reaching 60% for advanced neoplasia [15]. This could be explained by the fact that the topographical distribution of different pathological features is not uniform throughout colorectal neoplasia with both malignant and benign areas coexisting. More recently, a Korean study found a histologic discrepancy of 47.2% with 29.7% of the lesions being underdiagnosed with biopsies, similar to our data. The most important predictor of histologic discrepancy and under diagnosis was a size of the polyp > 10 mm [16]. These results underline the sampling bias existing with biopsy forceps due to heterogeneity of the lesion that is even more the case for larger lesions such as in our work. Moreover, when deep invasive adenocarcinoma was diagnosed in the resected specimen, biopsies did not allow the evaluation of the depth of invasion and was unable to distinguish intramucosal and superficial invasive adenocarcinoma from deep invasive adenocarcinoma. NTB and TB reported adenocarcinoma with “infiltrating aspect” in respectively 6 and 8 cases over 19 deep invasive adenocarcinomas, but as the depth of invasion could not be specified, it was not discriminant to choose the best resection technique. Furthermore, the sensibility of NTB and TB was only 31.6 and 42.1% respectively to distinguish deep invasive adenocarcinoma from other lesions whereas it reached 94.7% with endoscopic characterization. Biopsies specimens correspond to superficial pieces of the lesion. As so they may miss deep invasive component covered by adenomatous glands. Moreover, it does not allow any measurement as biopsies are not orientated specimens. Therefore, because of the high risk of under treatment, biopsies are not reliable to choose the resection technique since it could misclassify patient’s colorectal lesions and lead to performing an insufficient resection technique, such as piece meal for superficial malignant lesions or ESD for deep invasive lesions. Furthermore, biopsies are not without any effect on the tissue sampled. Fukunaga et al. demonstrated an increased rate of submucosal fibrosis in lesions who did undergo biopsy sampling with 20.6% of the lesions with fibrosis compared to 11.0% in lesions who did not. Even if, it must be noticed that in this Japanese study, there was no impact of preoperative biopsy as regard to ESD outcomes (procedure time, perforation, delayed bleeding) [17], a recent metanalysis demonstrated that fibrosis was associated with a higher risk of perforation with and odds ratio (OR) of 2.90 [18]. Prior biopsies have been associated with an increased risk of inability to perform complete resection of large nonpedunculated colorectal lesions (OR 0.24) and an increased risk of recurrence (OR 11.5) [19].

Nevertheless, several biases concerning biopsies could have affected the results of our study. First, performing biopsies after the end of the procedure as it was planned in our protocol could have induced a bias although our results concerning biopsies are consistent with other studies mentioned above. Although pathologists declare that they did not notice any difference in the quality of study samples compared to others, no specific analysis was conducted to demonstrate it in a sure way. Then, the zone of interest of the lesion selected to perform TB was identified by using endoscopic characterization itself. This could have induced a bias since one of the aims of this study was to compare TB and endoscopic characterization. Anyway, endoscopic criteria used in this study are widely supported by European and international guidelines [2]. Moreover, differences between endoscopic characterization and biopsies reported in this work are even more relevant when using NTB, whereas NTB were randomly performed on the piece and do not rely on endoscopic characterization.

Endoscopic characterization was associated with an increased risk of over treatment, especially with the CONECCT classification with 45.5% of over treatment whereas no case of over treatment was reported with biopsies. According to endoscopic characterization, 14/88 (15.9%) to 36/88 (40.9%) adenomas would have been resected with a R0 intent (by ESD) whereas a resection without R0 intent (piece meal with EMR) would have been sufficient. However, only two (2.8%) cases of severe adverse events, including one case of severe bleeding and one case of perforation leading to surgery, occurred in this study by performing ESD instead of EMR. A recent metanalysis of 49 studies demonstrated a similar risk of adverse event between EMR and ESD for laterally spreading tumors with a higher perforation rate in patients treated by ESD but lower risk of bleeding compared to EMR [20]. Perforations during ESD can be easily managed and led to surgery in only few cases [21]. On the contrary the risk of recurrence is higher when a lesion is removed by endoscopic piecemeal resection compared to ESD (0% with R0 ESD compared to about 20% with piecemeal, which can decrease to around 5% with thermal ablation of the post-resection mucosal margin defect [22]) and pathological analysis is easier when the lesion is removed en bloc instead of piecemeal. Indeed, in the latter, the loss of orientation induced by the fragmentation of the specimen and the formol induced shrinkage, led to a global loss of information. So even if over treatment would have been proposed more often for lesions characterized endoscopically, this would have been non deleterious for a large majority of them. A medico-economic study by our group is being carried out to determine the best cost-effectiveness strategy between ESD and EMR for lesions for which a resection by EMR would have been sufficient.

However, the increased risk of over treatment in the endoscopic characterization group could lead to propose surgery whereas endoscopic resection should be preferred. In our study, based on endoscopic characterization, 4/88 (4.5%) adenocarcinomas (one intramucosal and three superficial invasive adenocarcinomas) would have been resected surgically whereas ESD should have been performed. However, in some selected cases with a small invasive component (CONECCT III, Sano IIIb or Kudo Vn), ESD was attempted successfully with a diagnostic intent, allowing to diagnose the 3 superficial invasive adenocarcinomas. It suggests that this subgroup is heterogeneous considering the risk of deep invasion and that further criteria must be identified to determine which lesions with an invasive component should benefit from ESD [23]. A recent study reported that for colorectal lesions resected by ESD with a diagnostic intent, in which a suspicion of deep invasive adenocarcinoma was suspected according to endoscopic characterization (CONECCT III, Sano IIIb, Kudo Vn), ESD was curative in 29.4% of patients and could be a valid option for a further 31.8% when the deep invasive component was ≤ 10 mm [24]. Such a strategy could be considered as the first-line therapeutic option to avoid surgical treatment, especially in patients with significant comorbidities considering the high comorbidity rates of colon surgery (16.4% overall and 14.7% for benign colonic neoplasms) and the significantly higher cost compared to endoscopic resection [25].

In addition, a supplementary analysis was conducted considering that the presence of cancer on biopsies, including intramucosal carcinoma, would lead to surgical resection, as it is sometimes the usual practice in non-expert endoscopists. With such a strategy, NTB and TB were associated with respectively 3/88 (3.4%) and 4/88 (4.6%) cases of over treatment, leading to unnecessary surgery for two intramucosal and two superficial invasive adenocarcinomas.

In conclusion, during a colonoscopy, predicting the precise histology of colorectal neoplasia to choose the best treatment strategy is not an exact science and improvements are needed. Biopsies-based strategies are unable to predict depth of cancer invasion and could be associated with a risk of under treatment of large colorectal lesions in near a third of the cases compared to only around 5% with endoscopic characterization. Endoscopic characterization could lead to over treatment, but mainly by ESD with low morbidity. Characterization with the CONECCT classification could decrease the risk of under treatment and avoid surgeries for non-malignant colorectal lesions. Other endoscopic criteria should be determined to better characterize colorectal lesions and to improve the best adapted treatment for each lesion.

## Abbreviations

CONECCT: Colorectal neoplasia classification to choose the treatment
EMR: Endoscopic mucosal resection
ESD: Endoscopic submucosal dissection
ESGE: European society of gastrointestinal endoscopy
HGD: High-grade dysplasia
LGD: Low-grade dysplasia
LST: Laterally spreading tumor
LVI: Lymphovascular invasion
NTB: Non-targeted biopsy
OR: Odds ratio
Se: Sensitivity
SFED: Société française d’endoscopie digestive
Sp: Specificity
SSL: Sessile serrated lesion
TB: Targeted biopsy
VCE: Virtual chromoendoscopy
WLI: Wight light imaging
